# Validation of a novel time-to-event nest density estimator on passerines: An example using Brewer’s sparrows (*Spizella breweri*)

**DOI:** 10.1371/journal.pone.0227092

**Published:** 2019-12-30

**Authors:** Kaitlyn M. Reintsma, Alan H. Harrington, Victoria J. Dreitz

**Affiliations:** 1 Avian Science Center, Wildlife Biology Program, University of Montana, Missoula, Montana, United States of America; 2 Animal and Rangeland Sciences, Oregon State University, Corvallis, Oregon, United States of America; University of Illinois at Urbana-Champaign, UNITED STATES

## Abstract

Human impacts on natural resources increasingly necessitate understanding of the demographic rates driving wildlife population trends. Breeding productivity in many avian species is the demographic parameter that primarily influences population fluctuations. Nest density is a vital component of breeding productivity despite the fact that it is most often inferred exclusively from nest success. Unfortunately, locating every nest in a given area to determine nest density is often not feasible and can be biased by measurement error. The availability of a nest to be detected and the probability it will be detected during nest searching are two prominent sources of measurement error. A time-to-event nest density estimator has been developed that, unlike standard distance sampling methods, accounts for availability and can use nest data from outside structured surveys routinely collected to assess nest success. Its application is currently limited to Anseriformes, so we evaluated the general applicability of the time-to-event estimator in the order Passeriformes. To do this, we compared estimates of nest detection rate and nest density from the time-to-event estimator to distance sampling methods for 42 Brewer’s sparrow (*Spizella breweri*) nests monitored in 2015. The time-to-event estimator produced similar but more precise nest detection and density estimates than distance sampling methods.

## Introduction

Increasing human impacts on global natural resources continues to escalate the risk of extinction for many wildlife populations [1]. The need to understand how populations are affected by these impacts has never been greater [2]. Relying solely on population trend to assess populations can be misleading without knowledge of the demographic rates (e.g., productivity, survival, and age; [3]). Different demographic rates vary in their influences on population trend, as some processes influence a population more profoundly than others. For example, productivity is a crucial demographic rate in the life history of many short-lived organisms that can substantially affect population trends [3,4].

In general, avian species, especially songbirds, are short-lived and therefore typically sensitive to changes in breeding [5] demographic rates. This sensitivity to conditions on their breeding grounds forces them to respond quickly to short-term changes in the environment [6]. Thus, avian species are frequently used to understand human impacts to the environment and establish ecosystem-level conservation policies [7]. Breeding productivity is most often inferred from nest success rates, or the proportion of nests with at least one offspring fledging successfully [8]. However, breeding productivity is informed by three main variables that may vary over space and time: clutch size, nest success, and nest density [9]. Using only nest success to inform breeding productivity can be misleading when making inferences about avian populations and their environment [10, 11]. For example, an area with low nest success may have high breeding productivity by supporting high nest density or vice versa (e.g., [12, 13]). Estimating additional components of breeding productivity allows greater insight into the mechanism(s) that lead to fluctuations in avian populations.

Nest density estimates for avian species are often calculated by simply dividing the number of nests found by the area surveyed [14]. However, the importance of accounting for individuals present but not detected through sampling design and analytical estimation of density or abundance is well recognized [15, 16]. Nest density is similar in that estimates should include both the counts of detected nests and imperfect detection. For nests, imperfect detection includes two sources of error 1) availability of nests and 2) perceptibility [17]. Availability of nests depends on all biological and environmental factors that cause conspecific individuals to initiate nesting and become inactive (i.e. fledge or fail) on different dates throughout the breeding season [18]. Thus, nests may not be available for detection if observers do not survey the specific area while the nest is active. Perceptibility is the observers’ ability to detect nests given the nests are available, which varies by their skills, training, and weather, along with the nesting habitat, nesting stage, and bird behavior associated with each nest [17, 19].

Currently, the most widely accepted method to account for imperfect detection in nest density estimation is distance sampling [14]. In practice it provides transparent nest density estimation using established and structured methods that require limited auxiliary data. Distance sampling estimates a detection probability using the distance from a known observer’s location on a transect line or point to the location of the discovered nest. That is, distance sampling requires systematic surveys to estimate nest density. Data from nests found opportunistically, nests found when conducting follow up nest monitoring visits, cannot be included in distance sampling analyses. Distance sampling also does not account for availability ([14]; e.g., [20, 21]) and its assumption of detecting all nests on the line or point with certainty is difficult to achieve, especially in rough terrain ([22, 9]; e.g., [23]).

As an alternative to distance sampling, Péron et al. [24] introduced a time-to-event nest density estimator (TNDE) that allows for more flexible data collection and accounts for bias introduced by both sources of imperfect detection: availability and perceptibility. TNDE is a modified state-space superpopulation capture-recapture approach that estimates the availability of each nest using nest survival probabilities, then derives nest detection rates. Perceptibility is estimated by the number of times the nest escaped detection based on nest initiation and discovery dates [24]. Thus, TNDE generates estimates of nest density based on the number of observed nests and non-observed nests, which are determined using estimates of availability and perceptibility. An advantage of TNDE is that data regularly collected to estimate nest success (e.g., nest fate, clutch completion date, date discovered) are used to estimate nest density. In addition, differences in survey methods and a lack of standardized surveying are allowed in superpopulation models [25]. Therefore, opportunistic nests can be included in TNDE analyses.

TNDE is potentially another useful nest density estimator, however, studies are needed to validate TNDE and identify its level of generalization across avian groups. To our knowledge, the validation of TNDE is limited to the original study species, Blue‐winged Teal (*Anas discors*), used to define the analytical model (however, see [26] for an example of use). Here, we evaluate TNDE using an avian species with different nesting ecology to the original study species, Brewer’s sparrow (*Spizella breweri*). We assess TNDE estimates by comparing nest detection and nest density between the TNDE and the widely accepted nest density estimation methodology, distance sampling. We predict TNDE will estimate slightly higher nest density because it estimates a superpopulation over time while distance sampling estimates a population at a singular point in time. Our results from the two methodologies provides insight into when it is more advantageous to use distance sampling, TNDE, or a combination of both methods.

## Methods

### Ethics statement

All work was carried out according to relevant national and international guidelines. Research was approved by the University of Montana Institutional Animal Care and Use Committee (permit number 0212).

### Data collection

This study was a collaborative effort with an ongoing study on passerine communities in sagebrush habitat near Roundup, Montana. Our nest surveys took place on public lands managed by the Bureau of Land Management and lands in private ownership with established access agreements. In 2015, we selected 7 study sites of 250 km^2^ where relatively high concentrations of Brewer’s sparrow nests (3–4 nests per study site) were found using minimal nest search effort in 2013 and 2014 (VJ Dreitz, University of Montana, unpubl. data). Subsequent analysis was on data from the overall study area (1,750 km^2^; 46° 37′ 8.42″ N, 108° 39′ 28.00″ W), which is the aggregation of all study sites. Adhering to distance sampling protocols, we systematically selected line transects of 500 m in length to nest search within each of the 7 study sites. Each distance sampling nest survey consisted of four or five transects spaced 100 m apart and staggered 50 m each subsequent survey. Two observers positioned 5 m from the transect line walked North or South on either side of each transect line while gently sweeping sagebrush 5 m to the East and West to flush adult Brewer’s sparrows from nests [27]. A total of 3 distance sample nest surveys were conducted on each of the 7 study sites from 21 May to 7 July, the peak Brewer’s sparrow nesting period [28]. In addition to the distance sampling nest surveys, opportunistic nests were located on the 7 study sites during nest monitoring visits and other research activities as part of the larger ongoing study.

The location of each nest was recorded, and each nest was marked with flagging at a distance of approximately 5 m in the four cardinal directions. All nests were monitored approximately every 3 days until nest fate was determined as either success, at least one nestling fledged [29], or failed. In the event we returned to a nest and the appropriate amount of time for nestling development to fledgling (approximately 8 days, [28]) had passed, the nest was assumed to be fledged if no sign of mortality was present at the nest site. We also recorded the number of eggs or nestlings and documented the nest with a photograph at each visit. The photos were used to determine hatch and clutch completion date for each nest by assessing the age of nestlings with a species-specific aging guide (KM Reintsma, University of Montana, unpubl. data) and back-calculating the anticipated hatch date, assuming an 11-day incubation period [28].

### Distance sampling

We estimated the nest density for all study sites using all possible key functions in the distance package [30] in program R. Distance sampling estimation requires information on 1) the perpendicular distance from the transect line to the object of interest and 2) effort in the form of distance surveyed for each transect [14]. For our final model we used a hazard rate function to model the probability of detection for analyses because both model selection using Akaike’s Information Criteria (AIC) and visual assessment indicated best fit.

### Time-to-event nest density estimator

We estimated the nest density for all study sites using all combinations of parameter variation in nest stage (i.e. incubation and nestling) in the R package developed for the TNDE, nestAbund [24]. A metric for effort is not incorporated in TNDE. It is assumed that the study area is visited daily and a high probability of discovering any given nest, which we achieved. The average length of incubation and nestling periods was obtained from a previous Brewer’s sparrow study as 11 days and 9 days respectively [28]. The TNDE has three general steps to develop the superpopulation estimate. First, TNDE estimates the probability of nest success or failure on any given date given the date of initiation (i.e. availability). Then TNDE estimates the probability of nest detection on the day it was found given the nest was available for detection (i.e. perceptibility). Finally, TNDE estimates the superpopulation using the number of nests found and the probability a nest was not detected because it was not available or observed [24]. Our final model allowed for variation in the probability of detection but not for nest survival between incubation and brooding based on AIC values.

## Results

**Fig 1:**
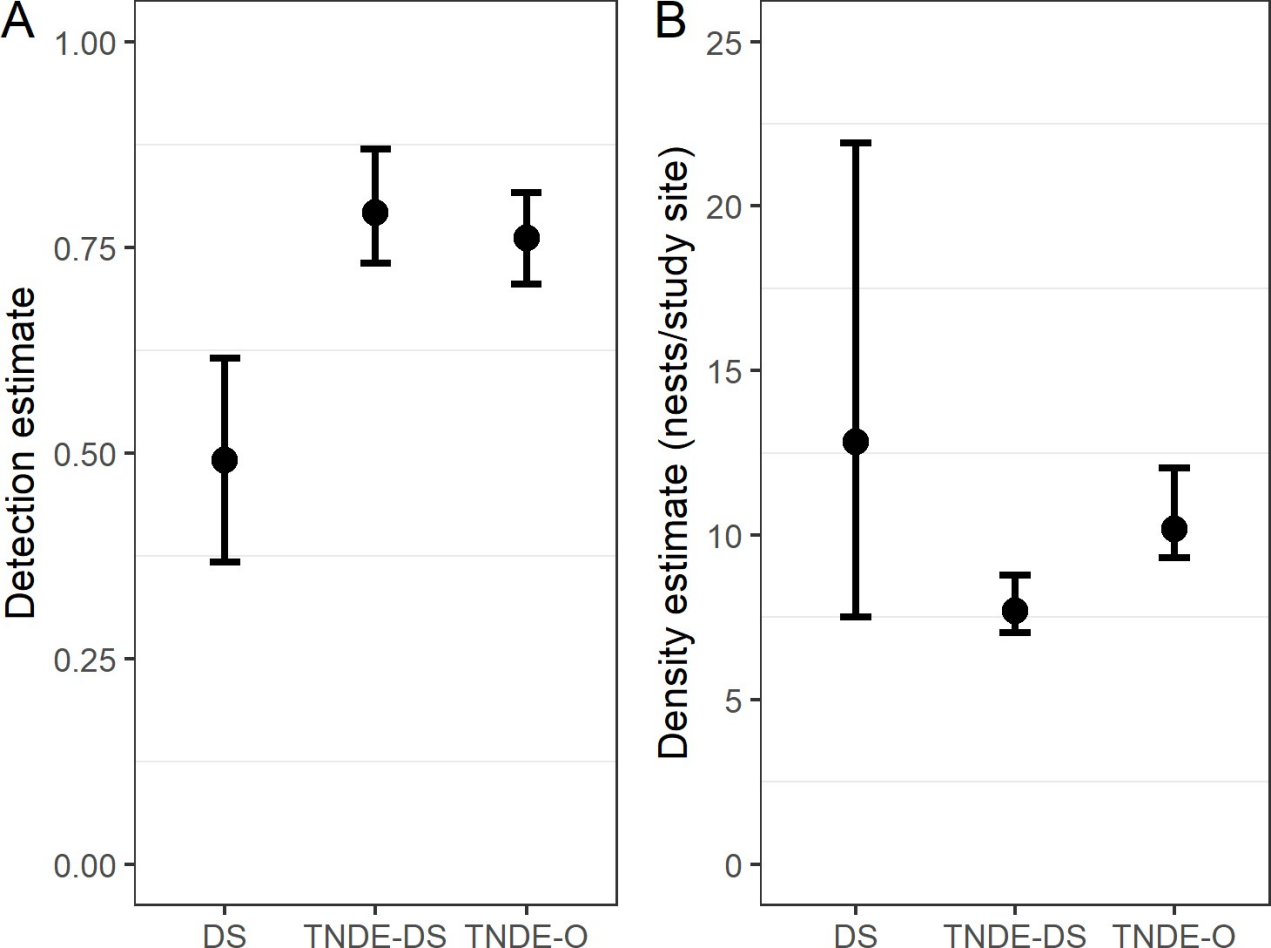
Detection and density estimates by analysis type. (A) Plots of the estimated detection probability and (B) average density of Brewer’s sparrow nests per study site with 95% confidence intervals for each estimation method.

We monitored a total of 68 nests; 42 (61.76%) nests were found using distance sampling surveys, and 26 (38.24%) nests were opportunistic. Based on data from the 42 nests found during distance sampling surveys, distance sampling estimates (DS) produced larger variance and lower probabilities for nest detection (DS; 0.49, 95% CI: 0.37–0.62) than TNDE estimates (TNDE-DS; 0.75, 95% CI: 0.61–0.80; (Fig 1A). The addition of data from opportunistic nests using TNDE (TNDE-O) resulted in smaller confidence intervals and more precision in the detection estimate by comparison (Fig 1A). Nest density estimates were slightly lower in descending order: DS (12.84 nests/250 km^2^ study site, 95% CI: 7.52–21.92 nests/study site), TNDE-O (10.18 nests/study site, 95% CI: 9.30–12.04 nests/study site), then TNDE-DS (Fig 1B). Thus, TNDE produced similar, but more precise estimates to DS when using only nests found with distance sampling surveys (TNDE-DS) and when incorporating all 68 nests monitored (TNDE-O).

## Discussion

Our study suggests that TNDE is an appropriate method to estimate nest density of Brewer’s sparrows when compared to distance sampling methods. TNDE detection rates and precision are increased over distance sampling estimates for Brewer’s sparrows (Fig 1A and 1B), which is advantageous if the estimates are accurate. The minor difference in point estimates and overlapping confidence intervals between the systematically collected data used in distance sampling estimate (DS) and TNDE estimate (TNDE-DS) suggests both estimates are similarly accurate (Fig 1B). The inclusion of opportunistic nest data in the TNDE analysis (TNDE-O) was more precise than estimates from DS. However, using only nests discovered through distance sampling (TNDE-DS) produced nest density estimates that are slightly lower and more precise than the TNDE-O that incorporates both kinds of data (Fig 1B).

Overall, both distance sampling and TNDE have appropriate uses in nest density estimation. When nest density is the singular parameter of interest, the TNDE may be inefficient when compared to distance sampling techniques because it requires more effort and time in the field to collect the data needed to inform the TNDE model (e.g., nest monitoring to determine fate). In such instances, distance sampling methods might be preferred because return visits to nests to estimate nest density are not necessary. Conversely, TNDE may likely be more useful than distance sampling when researchers are interested in gathering data to inform nest success or systematic surveys are inefficient. For example, many raptor nests are highly dispersed or within hazardous terrain in which distance sampling methods are infeasible (e.g., [23]). In these instances, TNDE is a potential alternative for nest density estimates, with the benefit of maximizing sample size and increasing the precision in the detection and density estimates through the addition of opportunistic nests. Lastly, nest density estimates can be made from combining distance sampling structured surveys and TNDE statistical approaches.

In the future, additional studies should validate the performance of TNDE on other avian species with different nest dispersal patterns (e.g., Hirundinidae), more complicated habitat (e.g., Icteridae), differing nesting ecology (e.g., Galliformes, Accipitriformes, and Charadriiformes), and other taxa (e.g., reptile hibernacula, mammal dens) to determine the full robustness of the TNDE estimator. In addition, while we did not include covariates that can influence nest detection and survival such as vegetation structure, TNDE allows for covariates and this aspect of the estimator needs further evaluation. Identifying the sample size and lowest detection probability at which the TNDE will reliably compute nest density estimates (and underlying estimates of nest success) is also essential. With further validation, TNDE could potentially be used with established datasets to determine nest density, which can provide a complete assessment of breeding productivity with minimal additional effort. These advantages afforded by TNDE could be especially beneficial for conservation efforts guided by avian fecundity.

## Supporting information

S1 File Datasets for TNDE and distance sampling analyses. This excel file contains the dataset used for the time-to-event nest density estimator analysis (S1A) and the dataset used for the distance sampling nest density estimation analysis (S1B).
(XLSX)
